# The impact of NHS based primary care complementary therapy services on health outcomes and NHS costs: a review of service audits and evaluations

**DOI:** 10.1186/1472-6882-9-5

**Published:** 2009-03-06

**Authors:** Lesley Wye, Deborah Sharp, Alison Shaw

**Affiliations:** 1 Academic Unit of Primary Health Care, University of Bristol, 25 Belgrave Road, Bristol, BS8 2AA, UK

## Abstract

**Background:**

The aim of this study was to review evaluations and audits of primary care complementary therapy services to determine the impact of these services on improving health outcomes and reducing NHS costs. Our intention is to help service users, service providers, clinicians and NHS commissioners make informed decisions about the potential of NHS based complementary therapy services.

**Methods:**

We searched for published and unpublished studies of NHS based primary care complementary therapy services located in England and Wales from November 2003 to April 2008. We identified the type of information included in each document and extracted comparable data on health outcomes and NHS costs (e.g. prescriptions and GP consultations).

**Results:**

Twenty-one documents for 14 services met our inclusion criteria. Overall, the quality of the studies was poor, so few conclusions can be made. One controlled and eleven uncontrolled studies using SF36 or MYMOP indicated that primary care complementary therapy services had moderate to strong impact on health status scores. Data on the impact of primary care complementary therapy services on NHS costs were scarcer and inconclusive. One controlled study of a medical osteopathy service found that service users did not decrease their use of NHS resources.

**Conclusion:**

To improve the quality of evaluations, we urge those evaluating complementary therapy services to use standardised health outcome tools, calculate confidence intervals and collect NHS cost data from GP medical records. Further discussion is needed on ways to standardise the collection and reporting of NHS cost data in primary care complementary therapy services evaluations.

## Background

To make informed decisions about the usefulness of complementary therapies, service users, clinicians and NHS commissioners need good quality information on the contribution complementary therapies can make to improving health outcomes and reducing NHS costs. Although there has been extensive debate on the best way to assess the impact of complementary therapy treatments on health outcomes [1-3], randomised controlled trials tend to dominate. Randomised controlled trials are conducted in tightly controlled experimental environments in which a particular intervention is targeted to a medically defined symptom (e.g. acupuncture for migraine headaches). When treatments are removed from this experimental context and integrated into the real world of healthcare service delivery, these tight controls disappear and local contextual factors may alter the impact of the treatments. Hence, in investigating the potential usefulness of complementary therapies as part of mainstream healthcare provision, research into the effectiveness of treatments and the impact of services is necessary.

To date, however, the majority of research has been into the therapeutic effectiveness of complementary therapy treatments, with approximately 1500 trial based papers published annually [4]. More recently, the cost effectiveness of complementary therapy treatments has become a focus. A review of 14 studies of complementary therapy treatments meeting quality criteria found that seven treatments were cost effective, including guided imagery, relaxation and potassium diets for cardiac patients and osteopathy and chiropractic for neck pain [5]. Another economic review of five complementary therapy treatments concluded that four treatments resulted in additional costs to the NHS compared to usual care, largely to cover the costs of the practitioner. They also found that the estimates of cost of the complementary therapy treatments compared favourably with other interventions approved for use in the NHS [6]. Nonetheless, although research evidence on the clinical and cost effectiveness of complementary therapy treatments is growing, we have less information on the impact of complementary therapy services on health outcomes or NHS costs.

One attempt to address this was a report by Christopher Smallwood and colleagues published in 2005 [7]. Drawing on three case sites where complementary therapy services were provided in NHS settings, the authors came to the conclusion that *[In the] majority of cases, specific conditions have improved, as have patients' general health and sense of well-being... [and] there seems to be good reason to believe that a number of CAM (complementary and alternative) treatments offer the possibility of significant savings in cost *[7].

Perhaps unsurprisingly, given the controversy surrounding NHS provision of complementary therapies, the credibility of this report was challenged [8]. Notwithstanding, these were possibly overly bold assertions, in light of the limited quantity and questionable quality of some of the case study data.

### Aim of this study

In a previous exercise, we collected evaluations of 25 complementary therapy services to identify the methodologies used to assess services [9]. In addition, we explored the relationship between evaluation content and methodology and NHS funding and found that a favourable report did not necessarily result in NHS funding. Subsequently, we interviewed NHS funders and found that although health outcome information was useful, information on the impact of complementary therapy services on NHS resource utilisation (e.g. GP consultations, prescription and hospital services) was necessary to inform commissioning decisions [10].

We have since continued to collect service evaluations and the purpose of this paper is to report on the data contained within this larger collection of documents. Specifically, our aim is to identify the potential impact of primary care complementary therapy services on health outcomes and NHS costs, as reported in complementary therapy service evaluations. The target audiences for this paper are NHS commissioners, who may be considering provision of complementary therapy services, and current and future providers of NHS based complementary therapy services, who can build on the experiences of colleagues conducting earlier evaluations.

## Methods

### Search strategy

Because the majority of complementary therapy services are located within primary care, we limited our review to this sector. We collected published and unpublished evaluations from November 2003 to April 2008. A rigorous, comprehensive searching strategy was devised including:

Contacting colleagues at the Foundation for Integrated Health, mid-Devon Primary Care Research Group and the Universities of Bristol, Sheffield, Thames Valley and Westminster, who had conducted evaluations and/or were networked to identify others who had.

Telephoning professional complementary therapy organisations e.g. Society of Homeopaths, British Council of Acupuncture, General Chiropractic Council, General Osteopathic Council.

Identifying potential studies from bibliographies of reports previously collected.

Searching the database of registered users for the SF36 and MYMOP questionnaires.

Searching PubCAM, AMED (Allied and Complementary Medicine) and Google Scholar.

Hand searching the archives of several journals including *Complementary Therapies in Medicine*, *Homeopathy *and *Acupuncture in Medicine*.

Search terms were: audit, general practice, primary care, complementary, alternative, homeopathy, acupuncture, evaluation and service. A full list of all evaluations located is available on request.

### Inclusion and exclusion criteria

We included documents if the service was located within England or Wales, was delivered by NHS clinicians or professional therapists and was situated in a NHS primary care setting. An exception was the inclusion of the Lewisham service [11]. Although outpatient hospital based, this evaluation was included because it was one of only two which employed a randomised controlled trial methodology and was similar to other primary care based services. We excluded evaluations if:

they reported throughput alone (e.g. numbers of patient seen)

they described solely the setting up of the service

the service setting was private, a charity or outside England or Wales

the service was part of an acute hospital department e.g. physiotherapists using acupuncture for pain

Because of the lack of high quality evaluations, no studies were excluded on methodological grounds.

### Data extraction and analysis

We devised a proforma to identify the type of information contained in the reports including health outcome tools (e.g. SF36, SF12, MYMOP, Glasgow Homeopathic Hospital Outcome Score, etc.) and NHS cost data (i.e. hospital, GP consultation or prescription costs). We then selected evaluations which collected health status data, using SF36 or MYMOP. These outcome tools were chosen because they were the most commonly used standardised health status questionnaires and so comparison across different services was easier.

The SF36 is a questionnaire which asks the service user to assess their health status in eight domains, including physical functioning, role physical, social functioning, pain, vitality, mental health, role emotional and general health [12]. For example, for 'physical functioning' respondents are asked to score a number of statements about their specific abilities to climb stairs or walk a mile while for 'role physical', respondents score statements about the extent of their ability to perform physical tasks generally. Although there is considerable debate about interpretation of SF36 scores, it is generally held that an improvement of 10 points or more indicates a strong effect (see  'norm based scoring and interpretation').

MYMOP asks the service user to identify and then rate the first and second priority symptoms that "bother" them the most, an activity affected by those symptoms and overall wellbeing on a scale of 0 to 6 [13]. In some cases, a profile score, which amalgamates the scores from symptoms 1 and 2, wellbeing and activity, is calculated. An improvement of 1 point or more is considered clinically significant (see ).

In addition to selecting evaluations with SF36 and MYMOP health status data, we also selected evaluations with extractable NHS cost data obtained from medical records. Once all relevant documents were identified, we then extracted details including:

number of service users

data collection time points

baseline and follow up health status scores

baseline and follow up rates and costs of prescriptions, GP consultations and hospital consultations

confidence intervals

p values.

If confidence intervals were missing and it was possible, we calculated the confidence intervals ourselves.

We gathered the results from individual service evaluations into outcome specific tables (i.e. SF36, MYMOP, prescriptions and GP consultations) and compared results across the services. For costs relating to use of hospital services, the data could not be combined into one table and so the data from the two relevant complementary therapy services are presented separately. We considered synthesizing the data for each table, but decided against this as the therapies offered, service models and ways of collecting the data differed considerably between sites.

## Results

In total, we collected 49 documents for 40 services. Further details about the methodology and content of the reports have been published previously [9]. Of the documents collected, we found 21 documents for 14 services contained extractable data on NHS costs and/or health status. Details of the services and evaluation documents are summarised in Additional file 1.

### Health status – SF36

Of the 14 services meeting our criteria, six administered and reported SF36 data that could be extracted (Additional file 2) [11,14-17]. Confidence intervals were available for four of the six service evaluations. Across the evaluations, four of the eight SF36 domains consistently have confidence intervals which do not cross zero for the average difference between baseline and follow up scores (role physical, social functioning, pain and vitality). This suggests that the complementary therapy services in this review have had a positive effect on the scores for the health status domains for these samples of service users. The pain scores showed the largest change. The fewest changes across these four services appear to have been made in role emotional, mental health and general health.

Of those using the SF36, only the Lewisham service also administered this questionnaire to a waiting list control group. The Lewisham service provided homeopathy, acupuncture and osteopathy delivered by professional therapists for over 20 different conditions. The baseline SF36 was administered before the first treatment and follow up occurred at the last session or three months after baseline (whichever came first). One hundred and seventy nine people in the treatment group and 151 in the control group completed baseline and follow up SF36 questionnaires. Results suggest a moderate to strong improvement for seven of the eight SF36 areas; only physical functioning showed no change [11].

### Health status – MYMOP

Of the 14 services included in the review, nine reported MYMOP data, but only seven provided extractable data (Additional file 3). In comparing the scores for the five services with confidence intervals, overall the first symptom identified by service users showed the greatest change followed by the second symptom. The average change in score was consistently greater than one, and in some cases it was closer to a two and half point difference. This suggests that these complementary therapy services had a substantial effect on health status scores, as measured by MYMOP, for these service users. Only the confidence intervals for the activity domain for the Sheffield service crossed zero (average difference 1.9, 95% CI -0.4 to 4.2), which suggests that the Sheffield complementary therapy service did not have a positive impact on the activity scores for this sample of service users. This may be understandable as service users were suffering from the menopause and symptoms do not tend to impact on activity levels.

### NHS costs

The quality and quantity of data on NHS costs was less robust or available than data for health status. Seven evaluations reported cost data extracted from GP medical records, one of which used randomised controlled trial methodology. Although all of the reports had methodological flaws, two were of especially poor quality (Newcastle [18] and St. Margaret's [19]). In these evaluations, a sub-sample of patients was identified (unclear as to how selected), relevant medical records were extracted and then the findings for the sub-samples were extrapolated across the entire service populations, resulting in guesstimates of potential savings. Nonetheless, as both of these evaluations justified further funding of these services by the NHS, and in the absence of better cost data, they are reported here.

A recurring methodological problem is that NHS cost data are less easily standardised than health status data. We found that the different evaluations used different ways to calculate costs. For example, prescription data was collected and analysed as average costs of prescriptions per month per patient, average number of prescriptions per month per patient, proportion of patients who reduced their number of prescriptions overall, total number of prescriptions and total cost savings of reduction in prescriptions by the entire sample. GP consultation data were more homogeneous in that all data were reported as consultation rates, but the time period varied between average consultation rates per patient per month, per six months or per year.

In looking at prescription costs, three out of six uncontrolled evaluations reported that service users reduced their prescriptions substantially by 57% (Coventry [20]), 45% (Glastonbury [21]) and 39% (Newcastle [18]). St. Margaret's reported potential savings of £8944. Results from the Impact evaluation suggested that there was no change in the number of prescriptions (change of 0.04, 95% CI -0.99 to 0.87) [16]. The prescription costs for service users of Get Well UK increased after using the service (average baseline cost per patient per month £3.24, 95% CI £1.80 to £4.80 and average follow up cost per patient per month £3.75, 95% CI £1.74 to £6.49) [22]. (Additional file 4)

In looking at GP consultation rates, three of the six uncontrolled evaluations reported that their sample of service users consulted their GPs about a third less often (Glastonbury [21] Newcastle [18] and Coventry [20]), while the St. Margaret's evaluation [19] found that service users consulted their GPs over two thirds less often. The results for the Impact service evaluation found that there was almost no change (change of 0.14, 95% CI -0.97, 1.83) [22]. Data from Get Well UK indicated that GP consultation rates amongst their sample of service users increased from an average of 0.5 per patient per month (95% CI 0.4, 0.7) at baseline to an average of 0.8 (95% CI 0.6 to 1.1) at follow up. Moreover, the Get Well UK evaluation suggested an increase in GP consultation costs per patient per month with an average baseline cost of £11.27 (95% CI £8.60, £13.90) and an average follow up cost of £17.53 (95% CI £11.40, £24.00) [22]. To put consultation rate data into context, the average practice consultation rate per listed patient per month in England was 0.44 in 2006 [23]. (Additional file 5)

The Get Well UK and Glastonbury reports provided data on secondary care consultations. The Get Well UK evaluation found that the rates of secondary care referrals and diagnostic tests combined per month were reduced (average combined of 1.38 at baseline to average combined of 0.70 at follow up), as were their corresponding costs (mean £112.64 at baseline to mean £64.72 at follow up) [22]. The Glastonbury evaluation found that usage of physiotherapy, x-rays, blood and urine, tests and consultant referrals were all reduced for a sub-sample of 41 patients with a total saving of over £2500 [21].

Only the Randomised Osteopathic Manipulation Study (ROMANS) collected NHS cost data for a control group. This was a pragmatic randomised controlled trial to evaluate a medical osteopathy service [24]. Two hundred and one patients with neck and back pain were randomised into two groups: usual GP care or medical osteopathy from a single GP practitioner. Service users in the active group received three to four medical osteopathy consultations. Medical record data on healthcare utilisation for 101 people in the usual care group and 86 in the medical osteopathy group were collected. Data for over twenty different NHS healthcare activities were collected, including rates for prescriptions, GP consultations and secondary care activities such as consultant and physiotherapy consultations. When calculating costs for all conditions suffered by the osteopathy service users and non-users, there was no difference between the medical osteopathy group and the control group (average total costs £22, 95% CI -£159, £142). Costs related to spinal pain were higher in the group using medical osteopathy than those who did not (average cost difference of £65, 95% CI £32, £155). This might be partly explained by the inclusion of the costs of the medical osteopathy consultations themselves [25]. (Table 1)

**Table 1:** NHS healthcare utilisation rates for ROMANS medical osteopathy service users and non-users for six months (during and after)

**Activity**	**Non-service users (SD)**	**Medical osteopathy service users (SD)**	Difference (95% Confidence Intervals*)
All GP contacts	3.26 (2.69)	3.16 (2.81)	**-0.10**

GP contacts for spinal pain	1.75 (2.22)	1.49 (2.0)	**-0.26**

All prescriptions	5.11 (7.41)	5.28 (8.62)	+0.17

Analgesic/non-steroidal anti-inflammatory drug prescriptions	1.3 (2.17)	1.21 (1.9)	**-0.09**

All consultant contacts	0.28 (0.62)	0.26 (0.49)	**-0.02**

Consultant contacts for spinal pain	0.09 (0.38)	0.06 (0.24)	**-0.03**

All physiotherapy	0.81 (1.96)	0.38 (1.76)	**-0.43**

Physiotherapy for spinal pain	0.73 (1.96)	0.36 (1.73)	**-0.37**

Average total healthcare costs	£307 (£687)	£328 (£564)	+£21 (-£142, £159)

Average total spine related costs	£64 (£90)	£129 (£283)	+£65 (£32, £155)

* 95% confidence intervals of the difference cannot be calculated as standard deviation not provided

Differences in bold = difference in favour of medical osteopathy group

SD = standard deviation

## Discussion

### Summary of key points

Few services collected data on health status using standardised health outcome tools and even fewer collected data on NHS costs. Of those that did, the quality of the evaluations was variable.

In comparing research into the effectiveness of complementary therapy *treatments *and the impact of complementary therapy *services *on health outcomes, we found that service evaluations were largely positive. All service evaluations collecting data on health status (SF36 or MYMOP) without a control group showed a substantial improvement in scores. When data were also collected for a control group (Lewisham), health status scores continued to demonstrate positive changes. With regard to the SF36, across evaluations both with and without a control group, the greatest changes were consistently found in role physical, social functioning, pain and vitality. Although more studies are needed, this suggests that NHS complementary therapy services may have an impact on health outcomes.

Data from complementary therapy service evaluations on NHS costs were much scarcer and less robust. Uncontrolled service evaluations found increases, decreases and no change in prescriptions and GP consultations. Both uncontrolled evaluations found decreases in secondary care usage. The only controlled study investigating the impact of a complementary therapy service on NHS costs (ROMANS) found that the medical osteopathy service made no impact on healthcare utilisation costs for all conditions. Costs associated only with spinal pain, which included the costs of the medical osteopathy consultations, were increased.

### Strengths and limitations

A strength of this study is that this is the first comprehensive attempt to collect and review the growing number of evaluations of NHS complementary therapy services in primary care. However, because of the scarcity of good quality data, we can draw few conclusions about the impact of these services on health status and NHS costs.

One limitation of this study is that very few evaluations met our selection criteria of reporting standardised health status or NHS cost data. A second limitation is that amongst those who did, there were gaps in the reporting of the data collection processes and inconsistencies across the evaluations that made comparison difficult e.g. varying data collection time points, different health outcome tools, prescriptions calculated as rates, costs and total savings etc. A third limitation is that only two service evaluations collected data for control groups. Control groups are used to demonstrate that any changes that have occurred can be attributed to the intervention (in this case a complementary therapy service) and would not have occurred anyway. This is necessary to assure some (scientifically minded) clinicians and Primary Care Trust managers of the potential impact of complementary therapies on health outcomes and NHS costs [26].

### Implications

Because NHS based complementary therapy services are often marginalised, face constant battles to secure funding [27] and have limited access to research expertise, those services that do carry out service evaluations deserve congratulations. Nonetheless, evaluations of NHS primary care complementary therapy services need greater rigour to provide better understanding of the impact these services can make on health outcomes and NHS costs. An earlier attempt to address this was the BESTCAM Delphi exercise which aimed to improve the *content *of complementary therapy service evaluations by identifying useful data collection items [28]. Our intention is to focus on improvements in the *process *of data collection and reporting.

**Figure 1:**
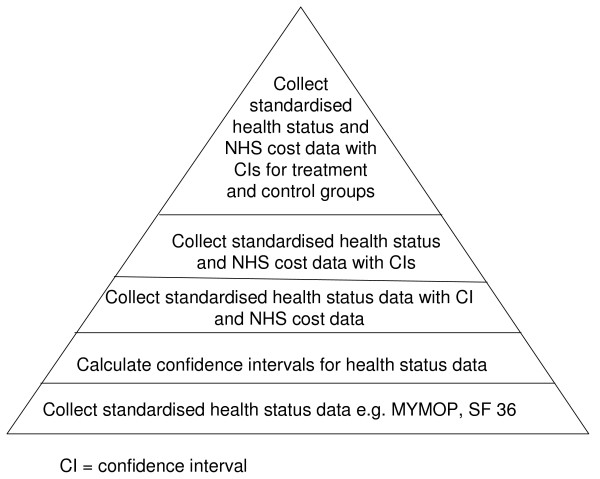
**Quality markers for evaluations of NHS primary care complementary therapy services**.

The following figure illustrates a suggested scale of quality markers for evaluations of complementary therapy services. (Figure 1) At a basic level, those evaluating complementary therapy services could collect data on health outcomes with standardised outcome tools such as MYMOP and SF36, rather than designing their own questionnaires. Although there are many such tools available, we found that MYMOP and SF36 were most commonly used in complementary therapy service evaluations. In comparing SF36 to MYMOP, the SF36 allows for better identification of the domains where complementary therapy services may score the largest improvement, but MYMOP is more patient oriented. Both of these are available without charge on the Internet (see  and ).

A further step in improving the quality of evaluations of NHS complementary therapy services would be the inclusion of confidence intervals around estimates. Confidence intervals provide valuable information on the range of values that might occur and give an indication of the strength of the impact of an intervention (in this case, a complementary therapy service). So, for example, for the first symptom for the CHIPs service [29] there was an average improvement of 1.9 for service users between baseline and follow up MYMOP scores on a six point scale. Using confidence intervals, we can say that we are 95% confident that the value of that difference within this population will fall somewhere between 1.5 and 2.3, which suggests a moderately strong impact. If a confidence interval crosses zero, this suggests that the service does not have an impact on improving the score for that domain. Although potentially daunting, confidence intervals are not difficult to calculate and instructions can be found in Additional file 6.

A further improvement in the quality of evaluations would be the collection of NHS cost data from GP medical records. This is a significant undertaking, as it requires obtaining permission to access medical records from GP surgeries (and possibly ethics approval see ), an understanding of medical terminology and extensive time. Furthermore, there is great variety in the way NHS cost data are collected as, unlike health status data, there are not standardised tools. However, the evaluations in this review showed a trend towards the calculation of GP consultation rates as average rates per patient over six or twelve months. Further research is needed into the optimum way of collecting and calculating prescription and secondary care data.

Once NHS cost data are collected, a further rung on the quality marker scale would be to calculate confidence intervals for cost data in addition to health status data.

Each of the first four stages on the quality marker triangle would require increasing confidence with research language and skills, although all of them could conceivably be undertaken with little or no academic involvement. However, the final step on the quality marker scale, to collect standardised health status and NHS cost data with confidence intervals for treatment and control groups, i.e. complementary therapy service users and non-users, would require significant engagement with academic researchers, possibly from a registered clinical trial unit (see ). But such an endeavour would also necessitate substantial outside funding. This could help explain why so few randomised controlled trials of complementary therapy services have taken place. Moreover, even if conducting randomised controlled trials were less challenging, we do not know the extent to which randomised controlled trials actually influence clinicians and NHS commissioners' decisions around complementary therapy service provision [10].

## Conclusion

In reviewing complementary therapy service evaluations, we found that uncontrolled health status data suggest that such services improve health outcome scores, but the data on the impact of these services on NHS costs are scarcer and inconclusive. Moreover, the overall quality of these evaluations was poor. To improve the quality of evaluations and increase understanding of the impact these services may have, we urge those evaluating complementary therapy services to use standardised health outcome tools, calculate confidence intervals and consider the collection of NHS cost data from GP medical records. Furthermore, discussion with the wider NHS healthcare community is needed on the optimum ways to standardise the collection and reporting of NHS cost data in evaluations of complementary therapy services.

## Competing interests

The authors declare that they have no competing interests.

## Authors' contributions

Funding for the study was obtained by AS and DS. The study was conceived by LW. The majority of the data were collected and analysed by LW, with assistance from AS and DS especially at the interpretative stages. LW drafted the manuscript and DS made substantive revisions.

## Pre-publication history

The pre-publication history for this paper can be accessed here:



## Supplementary Material

Additional file 1 **Supplementary table one.** Evaluations of NHS based primary care complementary therapy services with standardised health outcome and NHS cost data Click here for file

Additional file 2 **Supplementary table two.** SF36 scores from six complementary therapy service evaluations without control groups Click here for file

Additional file 3 **Supplementary table three.** MYMOP scores for seven service evaluations without control groups Click here for file

Additional file 4 **Supplementary table four. **Changes in prescriptions identified in six service evaluations without control groups Click here for file

Additional file 5 **Supplementary table five.** Changes in GP consultation rates identified in service evaluations without control groups Click here for file

Additional file 6 **How to calculate confidence intervals** Click here for file

## References

[B1] Vickers A, Cassileth B, Ernst E, Fisher P, Goldman P, Jonas W (1997). How should we research unconventional therapies?. International Journal of Technology Assessment in Health Care.

[B2] Verhoef M, Lewith G, Ritenbaugh C, Boon H, Fleishman S, Leis A (2005). Complementary and alternative medicine whole systems research: Beyond identification of inadequacies of the RCT. Complementary Therapies in Medicine.

[B3] Walach H, Falkenberg T, Fonnebo V, Lewith G, Jonas W (2006). Circular instead of hierarchical: methodological principles for the evaluation of complex interventions. BMC Medical Research Methodology.

[B4] Barnes J, Abbot N, Harkness E, Ernst E (1999). Articles on Complementary Medicine in the Mainstream Medical Literature: An Investigation of MEDLINE, 1966 Through 1996. Archives of Internal Medicine.

[B5] Herman P, Craig B, Caspi O (2005). Is complementary and alternative medicine (CAM) cost-effective? a systematic review. BMC Complement Altern Med.

[B6] Canter P, Coon Thompson J, Ernst E (2005). Cost effectiveness of complementary treatments in the United Kingdom: systematic review. BMJ.

[B7] Smallwood C (2005). The role of complementary and alternative medicine in the NHS.

[B8] Ernst E (2006). The 'Smallwood Report': method or madness?. British Journal of General Practice.

[B9] Wye L, Shaw A, Sharp D (2006). Evaluating complementary and alternative therapy services in primary and community care settings: A review of 25 service evaluations. Complementary Therapies in Medicine.

[B10] Wye L, Shaw A, Sharp D (2008). Designing a 'NHS friendly' complementary therapy service: a qualitative case study. BMC Health Services Research.

[B11] Richardson J (2001). Developing and evaluating complementary therapy services: part 2. Examining the effects of treatment on health status. Journal of Alternative and Complementary Medicine.

[B12] Garratt A, Ruta D, Abdalla M, Buckingham J, Russell I (1993). The SF36 health survey questionnaire: an outcome measure suitable for routine use within the NHS?. BMJ.

[B13] Paterson C (1996). Measuring outcomes in primary care: a patient generated measure, MYMOP; compared with the SF36 health survey. BMJ.

[B14] Hotchkiss J (1995). Liverpool Centre for Health. The first year of a service offering complementary therapies on the NHS – Observation Report Series no.25.

[B15] Thomas K, Harper R (1999). GP-based purchasing of osteopathy and chiropractic: an evaluation of a pilot scheme, 1996–1998.

[B16] Kelly S (2005). Untitled report.

[B17] Brown C (1995). Spiritual healing in a general practice: using a quality of life questionnaire to measure outcome. Complementary Therapies in Medicine.

[B18] Solomon D (2003). Complementary Therapy Pilot. Newcastle Primary Care Trust & Newcastle West Gate.

[B19] Christie E, Ward A (1996). A report on the NHS practice based homeopathy project: analysis of effectiveness and cost of homeopathic treatment within a GP practice at St. Margaret's Surgery.

[B20] Slade K, Chohan B, Barker P (2004). Evaluation of a GP practice based homeopathy service. Homeopathy.

[B21] Hills D, Welford R (1998). Complementary therapy in general practice: an evaluation of the Glastonbury Health Centre Complementary Medicine Service.

[B22] Robinson N, Donaldson J, Watt H (2006). Auditing outcomes and costs of integrated complementary medicine provision – the importance of length of follow up. Complementary Therapies in Clinical Practice.

[B23] Hippisley-Cox J, Fenty J, Heaps M (2008). Trends in consultation rates in GP practices 1995–2006 analysis of the QRESEARCH database.

[B24] Williams N, Wilkinson C, Russell I, Edwards R, Hibbs R, Linck P (2003). Randomized osteopathic manipulation study (ROMANS): pragmatic trial for spinal pain in primary care. Family Practice.

[B25] Williams N, Edwards R, Linck P, Muntz R, Wilkinson C, Russell I (2004). Cost utility analysis of osteopathy in primary care: results from a pragmatic randomized controlled trial. Family Practice.

[B26] Wye L, Shaw A, Sharp D (2009). Patient choice and evidence based decisions: the case of complementary therapies. Health Expectations.

[B27] Luff D, Thomas K (1999). Models of complementary therapy provision in primary care.

[B28] Wilkinson J, Peters D, Donaldson J (2004). Guidelines for developing Broad Evidence Synthesis topic for complementary and alternative medicine reports (BESTCAM reports).

[B29] Chaplin S (2005). CHIPs annual report 2004–2005.

[B30] Robertson F (2005). Impact Annual Report 2005. Nottingham.

[B31] Walters C, Batty J (2003). North Kirklees PCT Homeopathy Service Pilot Project.

[B32] Thomas K, Strong P, Luff D (2001). Complementary Medicine service in a community clinic for patients with symptoms associated with the menopause: outcome study & service evaluation.

[B33] Relton C, Weatherley Jones E (2005). Homeopathy service in a National Health Service community menopause clinic: audit of clinical outcomes. Journal of British Menopause Society.

[B34] Robinson N (2005). Does it work? A pilot project investigating the integration of complementary medicine into primary care.

[B35] Peters D, Andrews H, Hills D (2005). Integrating complementary medicine into primary care – an audit of five month referrals to the Get Well UK complementary therapy service in South Islington.

