# Supplementary material for: The impact of NHS based primary care complementary therapy services on health outcomes and NHS costs: a review of service audits and evaluations
Source: BMC Complement Altern Med. 2009 Mar 6;9:5. doi: 10.1186/1472-6882-9-5 (PMC2667472; doi:10.1186/1472-6882-9-5)
Supplement: Additional file 1 — Supplementary table one. Evaluations of NHS based primary care complementary therapy services with standardised health outcome and NHS cost data [file 1472-6882-9-5-S1.doc]

Table 1 Evaluations of NHS based primary care complementary therapy services with standardised health outcome and NHS cost data

| **Name of service** | **Therapies offered** | **Health outcome tool** | **No. matched returns** | **Time point baseline** | **Time point follow up** | **NHS cost data collected** | **Number medical records** | **Controlled study** | |
| --- | --- | --- | --- | --- | --- | --- | --- | --- | --- |
| Impact [16,30] | Acupuncture  Homeopathy  Chiropractic | SF36  MYMOP | 54  85 | “pre-treatment” | “end of treatment” | Prescriptions and  GP consultations | 28 | No | |
| Lewisham [11] | Acupuncture  Homeopathy  Osteopathy | SF36 | 179 (case)  151 (cntrl) | “prior to treatment” | “following treatment” (case)  3 months post baseline (control) | None | 0 | Yes | |
| Liverpool [14] | Acupuncture  Homeopathy  Osteopathy  Massage  Counselling | SF36 | 69 | Initial consultation | 8 weeks | None | 0 | No | |
| GP purchasing [15] | Osteopathy  Chiropractic | SF36 | 312 | At referral | 4 months | None | 0 | No | |
| Westcourt [17] | Spiritual healing | SF36 | 32 | “before 1^st^ session” | 8 weeks | None | 0 | No | |
| Coventry [20] | Homeopathy | MYMOP | 80 | Initial consultation | 6 months before & after 1^st^ consultation | Prescriptions  Consultations | 49  94 | No | |
| CHIPs [29] | Acupuncture  Homeopathy  Osteopathy  Massage  Reflexology  Nutrition + 4 others | MYMOP | 67 | Initial consultation | Final consultation | None | 0 | No | |
| North Kirklees [31] | Homeopathy | MYMOP | 65 | “before treatment commenced” | Final consultation | None | 0 | No | |
| Sheffield Menopause [32,33] | Homeopathy  Reflexology | MYMOP | 54 | At referral | Final consultation | None | 0 | No | |
| Glastonbury [21] | Acupuncture  Homeopathy  Osteopathy  Massage  Herbal med. | MYMOP | 114 | At referral | Final consultation | Prescriptions, GP consultations, hospital referrals and tests | 41 | No | |
| Get Well UK [22,34,35] | Acupuncture  Homeopathy  Osteopathy  Massage  Aromatherapy | MYMOP | 81 | Initial consultation | Final consultation | Prescriptions, GP consultations and hospital referrals | 33 | No | |
| Newcastle [18] | Acupuncture  Homeopathy  Osteopathy  Chiropractic  Massage  Shiatsu | Locally devised survey | Not known | Not known | Not known | Prescriptions and GP consultations | 70 | | No |
| St. Margaret’s [19] | Homeopathy | Locally devised survey | 133 | Not known | Not known | Prescriptions, GP consultations and hospital referrals | 24 (10% of service users) | | No |
| ROMANS [24,25] | Medical osteopathy | SF12 | 70 (case)  68 (cntrl) | Before randomisation | 2 months after starting treatment | Prescriptions, GP consultations, hospital consultations, admissions and tests | 101 (case)  86 (cntrl) | | Yes |
