# Supplementary material for: The impact of NHS based primary care complementary therapy services on health outcomes and NHS costs: a review of service audits and evaluations
Source: BMC Complement Altern Med. 2009 Mar 6;9:5. doi: 10.1186/1472-6882-9-5 (PMC2667472; doi:10.1186/1472-6882-9-5)
Supplement: Additional file 2 — Supplementary table two. SF36 scores from six complementary therapy service evaluations without control groups [file 1472-6882-9-5-S2.doc]

Table 2 SF36 scores from six complementary therapy service evaluations without control groups

| **SF36 domain** | **Evaluation** | **N** | **Baseline (SD)** | **Follow up (SD)** | **Difference (SD)** | **Difference time points** | **95% Confidence Interval** | **p value** |
| --- | --- | --- | --- | --- | --- | --- | --- | --- |
| Physical | Impact | 54* | 73.4 (20.4) | 80.6 (21.4) | 7.1 (16.6) | NK | 2.7, 11.5 | 0.02 |
| functioning | Lewisham | 179* | 53.5 (30.3) | 56.8 (31.9) | 3.4 | 6 – 12 wks | 0.6, 6.1 | 0.02 |
|  | Liverpool | 69* | 50 | 53 | 3 | 8 weeks | -1.8, 7.6 | 0.05 |
|  | Glastonbury | 93 | 68.1 | 72.9 | 4.8 | NK | ---------- | 0.001 |
|  | GP purchasing | 312 | ------- | -------- | 5.9 (19.5) | 16 weeks | 2.3, 9.5 | 0.000 |
|  | Westcourt | 32 | 70.2 (20.1) | 73.4 (22.7) | 3.2 | 8 weeks | ---------- | NS |
| Role physical | Impact | 54* | 50 (41.6) | 76.4 (30.3) | 26.4 (44.1) | NK | 14.6, 38.2 | 0.001 |
|  | Lewisham | 179* | 22.3 (33.4) | 44.7 (42.3) | 22.4 | 6 – 12 wks | 15, 29.9 | 0.000 |
|  | Liverpool | 69* | 26 | 35 | 9 | 8 weeks | 0.2, 18.3 | 0.06 |
|  | Glastonbury | 93 | 32.5 | 49.9 | 17.4 | NK | ---------- | 0.001 |
|  | GP purchasing | 311 | - | - | 27.1 (47.4) | 16 weeks | 21.8, 32.3 | 0.000 |
|  | Westcourt | 31 | 21.8 (30.1) | 47.6 (42.0) | 25.8 | 8 weeks | ---------- | <0.01 |
| Role | Impact | 54* | 52.4 (43.8) | 73.3 (39.5) | 20.9 (47.9) | NK | 8.1, 33.7 | 0.01 |
| emotional | Lewisham | 179* | 45.4 (45.0) | 60.5 (44.7) | 15.1 | 6 – 12 wks | 5.7, 24.6 | 0.002 |
|  | Liverpool | 69* | 41 | 48 | 7 | 8 weeks | -4.1, 17.4 | 0.2 |
|  | Glastonbury | 224 | 53.6 | 65.1 | 11.5 | NK | ---------- | 0.05 |
|  | GP purchasing | 308 | ----------- | ---------- | 9.0 (43.3) | 16 weeks | 4.2, 13.9 | 0.000 |
|  | Westcourt | 32 | 32.3 (38.3) | 54.2 (43.8) | 21.9 | 8 weeks | ---------- | <0.05 |
| Social | Impact | 54* | 66.1 (25.1) | 78.9 (23.7) | 12.7 (32.8) | NK | 4.0, 21.5 | 0.03 |
| functioning | Lewisham | 179* | 50.0 (30.5) | 62.8 (32.3) | 12.8 | 6 – 12 wks | 7.1, 18.5 | 0.000 |
|  | Liverpool | 69* | 55 | 62 | 7 | 8 weeks | 1.2, 13.9 | 0.02 |
|  | Glastonbury | 224 | 62.6 | 73.6 | 11 | NK | ---------- | 0.001 |
|  | GP purchasing | 321 | ----------- | --------- | 12.1 (27.2) | 16 weeks | 9.1, 15 | 0.000 |
|  | Westcourt | 33 | 52.1 (28.7) | 66.4 (26.5) | 14.3 | 8 weeks | ---------- | <0.01 |
| Pain | Impact | 54* | 47.8 (26.1) | 75 (21) | 27.2 (28.3) | NK | 19.7, 34.7 | <0.001 |
|  | Lewisham | 179* | 39.1 (27.9) | 52 (30.1) | 12.9 | 6 – 12 wks | 8.4, 17.4 | 0.000 |
|  | Liverpool | 69* | 40 | 47 | 7 | 8 weeks | 3.3, 12.9 | 0.000 |
|  | Glastonbury | 224 | 43.1 | 53.8 | 10.7 | NK | -------- | 0.001 |
|  | GP purchasing | 320 | ---------- | ---------- | 23.8 (26.9) | 16 weeks | 20.8, 26.7 | 0.000 |
|  | Westcourt | 32 | 44.6 (23.8) | 56.8 (26.2) | 12.2 | 8 weeks | ---------- | <0.05 |
| Vitality | Impact | 54* | 47 (17) | 58.1 (19.2) | 11.1 (22.7) | NK | 8, 14.2 | 0.006 |
|  | Lewisham | 179* | 34.8 (19.9) | 43.4 (22.8) | 8.6 | 6 – 12 wks | 4.7, 12.5 | 0.000 |
|  | Liverpool | 69* | 40 | 46 | 6 | 8 weeks | 0.6, 10.4 | 0.03 |
|  | Glastonbury | 224 | 41.9 | 48.3 | 6.4 | NK | ---------- | 0.005 |
|  | GP purchasing | 315 | ---------- | ------------ | 8.5 (19.1) | 16 weeks | 6.4, 10.7 | 0.000 |
|  | Westcourt | 32 | 32.5 (18.8) | 52.7 (19.8) | 20.2 | 8 weeks | ---------- | <0.001 |
| Mental health | Impact | 54* | 62.6 (19.7) | 72.9 (18) | 10.2 (20.4) | NK | 4.8, 15.6 | 0.005 |
|  | Lewisham | 179* | 55.0 (22.5) | 60.9 (22.6) | 5.9 | 6 – 12 wks | 2.3, 9.4 | 0.001 |
|  | Liverpool | 69* | 57 | 62 | 5 | 8 weeks | -0.1, 9.3 | 0.06 |
|  | Glastonbury | 224 | 59.5 | 67 | 7.5 | NK | --------- | 0.001 |
|  | GP purchasing | 317 | -------- | ---------- | 5.3 (16.7) | 16 weeks | 3.5, 7.1 | 0.000 |
|  | Westcourt | 32 | 49.6 (19.5) | 63.5 (22.0) | 13.9 | 8 weeks | ---------- | <0.001 |
| General | Impact | 54* | 57.9 (19.4) | 66.7 (20.9) | 8.9 (21.6) | NK | 3.1, 14.7 | 0.021 |
| health | Lewisham | 179* | 45.1 (23.1) | 50.6 (24.3) | 5.5 | 6 – 12 wks | 2.3, 8.7 | 0.001 |
|  | Liverpool | 69* | 53 | 54 | 1 | 8 weeks | -2.3, 6.1 | 0.3 |
|  | Glastonbury | 224 | 58.4 | 60.9 | 2.5 | NK | ---------- | NS |
|  | GP purchasing | 308 | ---------- | --------- | 0.5 (15.1) | 16 weeks | 6.4, 10.7 | 0.5 |
|  | Westcourt | 30 | 48.7 (20.9) | 55.4 (22.0) | 6.7 | 8 weeks | ---------- | <0.05 |

---------- data not provided or data not obtainable

*exact numbers for matched returns for each SF domain not given

NK = not known

NS = not significant
