# Supplementary material for: The impact of NHS based primary care complementary therapy services on health outcomes and NHS costs: a review of service audits and evaluations
Source: BMC Complement Altern Med. 2009 Mar 6;9:5. doi: 10.1186/1472-6882-9-5 (PMC2667472; doi:10.1186/1472-6882-9-5)
Supplement: Additional file 3 — Supplementary table three. MYMOP scores for seven service evaluations without control groups [file 1472-6882-9-5-S3.doc]

Table 3 MYMOP scores for seven service evaluations without control groups

| **MYMOP domain** | **Evaluation** | **N** | | **Baseline (SD)** | **Follow up (SD)** | **Difference (SD)** | **Time** | **95% Confidence Interval** | **p value** |
| --- | --- | --- | --- | --- | --- | --- | --- | --- | --- |
| 1^st^ symptom | Coventry | 80 | | --------- | --------- | 2.5 | Avg. 134 days | 2.1, 2.9 | <0.0001 |
|  | CHIPs | 67 | | 4.6 (1.3) | 2.7 (1.6) | 1.9 (1.8) | Varies | 1.5, 2.3 | --------- |
|  | N Kirklees | 65 | | 4.5 | 2.2 | 2.3 | Varies | 1.9, 2.8 | <0.001 |
|  | Impact | 85* | | 4.3 (1.1) | 1.6 (1.2) | 2.6 (1.7) | Varies | 2.2, 3.0 | <0.001 |
|  | Sheffield | 54 | | 5.0 | 2.6 | 2.3 (1.9) | varies | 1.8, 2.8 | <0.001 |
|  | Glastonbury | 114 | | 4.8 | 3.0 | 1.8 | NK | -------- | --------- |
|  | Get Well UK | 81 | | 4.7 | 2.6 | 2.1 | Varies | -------- | --------- |
| 2^nd^ symptom | Coventry | 55 | | --------- | --------- | 2.5 | Avg. 126 days | 2.0, 3.0 | <0.0001 |
|  | CHIPs | 49 | | 3.8 (1.6) | 2.5 (1.6) | 1.4 (2.0) | varies | 0.9, 2.0 | --------- |
|  | N Kirklees | --- | | ------------ | --------- | ---------- | ---------- | ---------- | ---------- |
|  | Impact | 85* | | 4.1 (1.3) | 1.7 (1.3) | 2.4 (1.5) | Varies | 1.9, 2.9 | <0.001 |
|  | Sheffield | 53 | | 4.8 | 2.9 | 2 (2.0) | Varies | 1.5, 2.5 | <0.001 |
|  | Glastonbury | 114 | | 4.3 | 2.8 | 1.5 | NK | -------- | -------- |
|  | Get Well UK | 81 | | 4.5 | 2.7 | 1.8 | Varies | -------- | -------- |
| Activity | Coventry | 63 | | --------- | --------- | 2.4 | Avg. 141 days | 2.0, 3.0 | <0.0001 |
|  | CHIPs | 54 | | 4.3 (1.5) | 3.0 (1.8) | 1.3 (2.0) | varies | 0.9, 1.9 | --------- |
|  | N Kirklees | ---- | | ---------- | ---------- | --------- | --------- | --------- | --------- |
|  | Impact | 85* | | 4.2 (1.1) | 2.1 (1.4) | 2.0 (1.6) | Varies | 1.5, 2.4 | <0.001 |
|  | Sheffield | 29 | | 4.6 | 2.7 | 1.9 (1.7) | Varies | -0.4, 4.2 | <0.001 |
|  | Get Well UK | 81 | | 4.7 | 2.6 | 2.1 | Varies | -------- | -------- |
| Wellbeing | Coventry | 73 | | --------- | --------- | 1.4 | Avg. 132 days | 1.0, 1.8 | <0.0001 |
|  | CHIPs | 67 | | 3.9 (1.2) | 2.6 (1.6) | 1.3 (2.0) | varies | 0.8, 1.8 | --------- |
|  | N Kirklees | 65 | | 3.6 | 2.0 | 1.6 | Varies | 1.2, 2.0 | <0.001 |
|  | Impact | 85* | | 3.6 (1.3) | 1.9 (1.2) | 1.7 (1.6) | Varies | 1.3, 2.1 | <0.001 |
|  | Sheffield | 39 | | 4.4 | 2.8 | 1.6 (1.9) | Varies | 1.0, 2.2 | <0.001 |
|  | Get Well UK | 81 | | 3.8 | 2.5 | 1.3 | Varies | -------- | --------- |
| Profile | CHIPs | 67 | 4.2 (1.2) | | 2.7 (1.4) | 1.5 (1.4) | varies | 1.1, 1.8 | --------- |
|  | Sheffield | 29 | 4.9 | | 2.7 | 2.1 (1.6) | Varies | 1.5, 2.7 | <0.001 |
|  | Get Well UK | 81 | 4.5 | | 2.8 | 1.7 | Varies | -------- | --------- |

--------- data not provided or data not obtainable

* exact number of matched returns not given for each MYMOP domain
