# Supplementary material for: The impact of NHS based primary care complementary therapy services on health outcomes and NHS costs: a review of service audits and evaluations
Source: BMC Complement Altern Med. 2009 Mar 6;9:5. doi: 10.1186/1472-6882-9-5 (PMC2667472; doi:10.1186/1472-6882-9-5)
Supplement: Additional file 4 — Supplementary table four. Changes in prescriptions identified in six service evaluations without control groups [file 1472-6882-9-5-S4.doc]

Table 4 Changes in prescriptions identified in six service evaluations without control groups

| **Evaluation** | **Therapies offered** | **Data collection details** | **No. re-cord** | **Data extraction time points** | **Baseline (95% CI)** | **Follow up (95% CI)** | **Change (95% CI)** | **Total savings in pounds** | **Direction of change** |
| --- | --- | --- | --- | --- | --- | --- | --- | --- | --- |
| **Get Well UK** [22] | Acupuncture  Homeopathy  Ostoeopathy  Massage  Aromatherapy | Average cost prescriptions per month per patient for referred condition | 33 | Pre 24 months Post average 5.7 months | Average £3.24  (£1.80, £4.80) | Average £3.75 (£1.74, £6.49) | +£0.51 | Not calculated | Increase |
| **Impact** [16] | Acupuncture  Homeopathy  Chiropractic | Average number of prescriptions per month per patient | 28 | Pre 16 months  Post 9 months | 0.41 (0,1.5) | 0.38 (0,1.28) | -0.04  (-0.99, 0.87) | Not calculated | No change |
| **Coventry** [20] | Homeopathy | Proportion of patients who reduced prescriptions  Total savings across sample | 49 | Pre 6 months  Post 6 months | Not given | Not given | 28 people reduced or stopped  (57% reduction) | £2807.30 | Decrease |
| **Glaston-bury** [21] | Acupuncture  Homeopathy  Osteopathy  Massage  Herbal med | Number of prescriptions per person per year#  Total savings across sample | 41 | Pre 12 months  Post 12 months | 2.15 (n=88) | 1.17 (n=48) | -0.98  (45% reduction) | £382.47 | Decrease |
| **Newcastle*** [18] | Acupuncture  Homeopathy  Osteopathy  Chiropractic  Massage  Shiatsu | Proportion of patients who reduced prescriptions  Total savings across sample | 70 | Pre 6 months  Post 6 months | Not given | Not given | 41 people stopped/ reduced  (39% reduction) | £520 | Decrease |
| **St Marg-aret’s*** [19] | Homeopathy | Total savings across sample | 24 | Pre 12 months  Post 12 months | Not given | Not given | Not given | £8944 | Decrease |

# Rates have been calculated from the data provided.

* Poor data
