# Supplementary material for: The impact of NHS based primary care complementary therapy services on health outcomes and NHS costs: a review of service audits and evaluations
Source: BMC Complement Altern Med. 2009 Mar 6;9:5. doi: 10.1186/1472-6882-9-5 (PMC2667472; doi:10.1186/1472-6882-9-5)
Supplement: Additional file 5 — Supplementary table five. Changes in GP consultation rates identified in service evaluations without control groups [file 1472-6882-9-5-S5.doc]

Table 5 Changes in GP consultation rates identified in service evaluations without control groups

| **Evaluation** | **Therapies offered** | **Details of data collection** | **No. re-cord** | **Data extraction time points** | **Baseline (95% CI)** | **Follow up (95% CI)** | **Change** | **Direction of change** |
| --- | --- | --- | --- | --- | --- | --- | --- | --- |
| **Get Well UK** | Acupuncture  Homeopathy  Osteopathy  Massage  Aromatherapy | Average rates and costs per patient per month | 33 | Pre 24 months Post average 5.7 months | 0.5 (0.4, 0.7)  £11.27 (£8.60, £13.90) | 0.8 (0.6, 1.1)  £17.53 (£11.40, £24.00) | +0.3  £6.26 | Increase |
| **Impact** | Acupuncture  Homeopathy  Chiropractic | Average rates per patient per month | 28 | Pre 16 months  Post 9 months | 0.89  (0.06, 3.87) | 0.75  (0.12, 2.66) | -0.14  (-1.0, 1.8) | No change |
| **Coventry** | Homeopathy | Average rates per patient per year | 94 | Pre 6 months  Post 6 months | 6.4 | 5.3 | -1.2 (0.4, 2)  (31%) | Reduction |
| **Glastonbury** | Acupuncture  Homeopathy  Osteopathy  Massage  Herbal med | Average rates per patient per year | 41 | Pre 12 months  Post 12 months | 3.1 | 2.2 | -0.9  (31%) | Reduction |
| **St Margaret’s*** | Homeopathy | Average rates per patient per six months | 24 | Pre 6 months  Post 6 months | 3.1 | 1.0 | -2.1  (71%) | Reduction |
| **Newcastle*** | Acupuncture  Homeopathy  Osteopathy  Chiropractic  Massage  Shiatsu | Average rates per patient per year | 70 | Pre 6 months  Post 6 months | 5.7 | 3.9 | -1.7  (31%) | Reduction |

* Poor data
