# Supplementary material for: The impact of NHS based primary care complementary therapy services on health outcomes and NHS costs: a review of service audits and evaluations
Source: BMC Complement Altern Med. 2009 Mar 6;9:5. doi: 10.1186/1472-6882-9-5 (PMC2667472; doi:10.1186/1472-6882-9-5)
Supplement: Additional file 6 — How to calculate confidence intervals [file 1472-6882-9-5-S6.doc]

Table 6 Calculating confidence intervals

To calculate confidence intervals, you need: the number of returns (known as n)

the mean (or average) difference between before and after scores and the standard deviation for each aspect (e.g. symptom 1, symptom 2, vitality, GP consultation rates, etc.). The mean difference and standard deviation scores can be generated through the use of any spreadsheet package, such as Excel. With standard deviations, the standard error can be calculated using the formula:

Standard error = Standard deviation/ the square root of n.

With the standard error, lowest and highest confidence intervals can then be calculated using the formula:

Confidence interval = mean – (1.96 x standard error) to mean = (1.96 x standard error).

For example, the Impact service evaluation generated 54 before and after returns for ‘pain’ (n=54), with a mean difference of 27.2 and a standard deviation of 28.3 (see Table 2). The square root of 54 is 7.35. Standard error = 28.2 / 7.35, which is 3.8. The lowest confidence interval can be calculated at 27.2 – (1.96 x 3.8), which is 19.7 and the highest confidence interval can be calculated at 27.2 + (1.96 x 3.8), which is 34.7. Therefore, we can say that the mean difference between before and after scores for pain in the Impact service found in this study was 27.2 and we are 95% confident that the true value of the mean difference lies between 19.7 and 34.7. This suggests that the service results in a moderate to strong improvement in pain scores.
